# Clinico-Pathogenic Similarities and Differences between Infection-Related Glomerulonephritis and C3 Glomerulopathy

**DOI:** 10.3390/ijms24098432

**Published:** 2023-05-08

**Authors:** Yukihiro Wada, Mariko Kamata, Ryoma Miyasaka, Tetsuya Abe, Sayumi Kawamura, Kazuhiro Takeuchi, Togo Aoyama, Takashi Oda, Yasuo Takeuchi

**Affiliations:** 1 Department of Nephrology, Kitasato University School of Medicine, 1-15-1 Kitasato, Minami-ku, Sagamihara 252-0374, Kanagawa, Japan; 2 Department of Nephrology and Blood Purification, Kidney Disease Center, Tokyo Medical University Hachioji Medical Center, Hachioji 193-0998, Tokyo, Japan

**Keywords:** infection-related glomerulonephritis, C3 glomerulopathy, membranoproliferative glomerulonephritis, nephritis-associated plasmin receptor, complement alternative pathway

## Abstract

Recently, the comprehensive concept of “infection-related glomerulonephritis (IRGN)” has replaced that of postinfectious glomerulonephritis (PIGN) because of the diverse infection patterns, epidemiology, clinical features, and pathogenesis. In addition to evidence of infection, hypocomplementemia particularly depresses serum complement 3 (C3), with endocapillary proliferative and exudative GN developing into membranoproliferative glomerulonephritis (MPGN); also, C3-dominant or co-dominant glomerular immunofluorescence staining is central for diagnosing IRGN. Moreover, nephritis-associated plasmin receptor (NAPlr), originally isolated from the cytoplasmic fraction of group A *Streptococci*, is vital as an essential inducer of C3-dominant glomerular injury and is a key diagnostic biomarker for IRGN. Meanwhile, “C3 glomerulopathy (C3G)”, also showing a histological pattern of MPGN due to acquired or genetic dysregulation of the complement alternative pathway (AP), mimics C3-dominant IRGN. Initially, C3G was characterized by intensive “isolated C3” deposition on glomeruli. However, updated definitions allow for glomerular deposition of other complement factors or immunoglobulins if C3 positivity is dominant and at least two orders of magnitude greater than any other immunoreactant, which makes it challenging to quickly distinguish pathomorphological findings between IRGN and C3G. As for NAPlr, it was demonstrated to induce complement AP activation directly in vitro, and it aggravates glomerular injury in the development of IRGN. A recent report identified anti-factor B autoantibodies as a contributing factor for complement AP activation in pediatric patients with PIGN. Moreover, C3G with glomerular NAPlr deposition without evidence of infection was reported. Taken together, the clinico-pathogenic features of IRGN overlap considerably with those of C3G. In this review, similarities and differences between the two diseases are highlighted.

## 1. Introduction

Postinfectious glomerulonephritis (PIGN) is one of the representative diseases presenting with acute glomerulonephritis (AGN) [1,2,3]. The typical presentation of PIGN includes hematuria, mild to moderate proteinuria, edema, and hypertension with a latency period following infection. Rapidly progressive GN also occurs rarely, and some patients progress to chronic kidney disease (CKD) [3]. During the past century, most cases of PIGN occurred in childhood and followed streptococcal infections, leading to it being called post-streptococcal acute glomerulonephritis (PSAGN) [2,3]. However, in the past three decades, the incidence of PSAGN has decreased, particularly in advanced countries, probably due to the improvement in the living environment and the appropriate administration of antibiotics [1,2,3]. Currently, the estimated annual incidence of PSAGN is 90–280 per 1,000,000 population [4,5]. On the other hand, in advanced countries in the modern era, the number of adult patients with AGN has been increasing. A significant proportion of adult AGN cases is associated with non-streptococcal infection, especially *Staphylococcus* infection [1,3]. At present, adult-onset AGN caused by *Staphylococcus* infection is as common as PSAGN, and such cases of AGN occur in elderly or immunocompromised patients, in whom it is a formidable issue because of their poor renal prognosis [1,2,3]. Moreover, non-streptococcal infections in adult AGN have heterogeneous infection sites and tend to be ongoing at the time of definitive diagnosis by renal biopsy (RB). Therefore, the term “infection-related glomerulonephritis (IRGN)” has been proposed [6] as the comprehensive concept including PSAGN and atypical types of adult-onset of AGN related to various infections (e.g., staphylococci, Gram-negative bacteria, various viruses, fungi, or protozoa) [7].

Complement 3 glomerulopathy (C3G) is a recently identified disease entity caused by acquired or genetic dysregulation of the complement alternative pathway (AP) [8,9,10,11,12]. The estimated annual incidence is 1–3 per 1,000,000 population [13]. Most patients with C3G show hematuria and nephrotic range proteinuria (NRP) [3]. The renal prognosis of C3G is not always good, and some patients progress to end-stage renal disease [13,14,15]. C3G can be further subdivided into dense deposit disease (DDD) and C3 glomerulonephritis (C3GN) [8,9,13,14,16,17,18]. In the clinical setting, C3GN is a rare kidney disease, thereby making diagnosis by physicians difficult. Furthermore, an expert in renal pathology may be required to confirm the diagnosis of C3G, since other glomerular lesions, such as IRGN or immune complex (IC) forms of membranoproliferative glomerulonephritis (MPGN), can mimic C3G. In particular, it is extremely difficult to morphologically differentiate C3GN from C3-dominant IRGN. Thus, a reliable biomarker for the differential diagnosis of C3G and IRGN is needed. Assays for complement components and gene analysis are the first step to determine the presence or absence of complement AP dysregulation in C3G patients. Positivity for glomerular nephritis-associated plasmin receptor (NAPlr) and related plasmin activity are anticipated as key diagnostic biomarkers for IRGN [19,20,21,22,23,24].

According to previous reports, a genetic variant in the complement AP factors or autoantibodies (C3 nephritic factor (C3NeF), anti-factor H (anti-FH) antibody, and anti-factor B (anti-FB) antibody) that induce a defect in the regulation of AP can be detected in approximately half of the cases with C3G [25,26,27,28]. It was recently reported that hypocomplementemia did not necessarily occur in all cases of C3G, despite the presence of underlying AP dysregulation [3]. Meanwhile, some patients with IRGN show persistent hypocomplementemia during their clinical course [3]. The association of inappropriate AP activation with the exacerbation of IRGN was emphasized in recent review articles [19,20]. Moreover, recent reports demonstrated a significant elevation of anti-FB autoantibodies in pediatric patients with PIGN compared to C3G [29,30]. C3G with glomerular NAPlr deposition without evidence of infection was noted [19]. Taken together, the clinico-pathogenic features of IRGN overlap considerably with those of C3G, which may lead clinicians in the field of nephrology to confuse them. In this review, the similarities and differences between these two diseases are highlighted, and the details of their clinico-pathogenic features are presented.

## 2. Method for Literature Search

PubMed was searched for articles in English using the search terms “GN”, “infection”, “C3G”, and “alternative pathway” in studies in humans. Review articles, observational studies, and case reports were selected as the available literature. We focused on articles published between 1 January 2012 and 31 December 2022. Relevant older articles were also retrieved by a manual search of reference lists.

Strong evidence in this field is generally limited, since no large prospective clinical studies have been performed due to the rarity of IRGN and C3G. In addition, a reliable animal model has not been established to date. Therefore, the contents of this review are mostly based on detailed evaluations of observational studies and case reports.

### 2.1. Pathomorphological Characteristics of IRGN and C3G

#### Differentiation between IRGN and C3G Based on the Diagnostic Criteria

In accordance with the previous review by Nasr et al., the diagnosis of IRGN is based on the constellation of several clinical features and pathological findings, as follows: (1) clinical or laboratory evidence of infection preceding or at the onset of GN; (2) depressed serum complement; (3) endocapillary proliferative and exudative GN on light microscopy (LM); (4) C3-dominant or co-dominant glomerular deposition on immunofluorescence (IF) microscopy; and (5) hump-shaped subepithelial deposits on electron microscopy (EM) [2,31,32]. They mentioned that fulfilling all five criteria is not necessary, and at least three of the above-described criteria are sufficient for a diagnosis of IRGN. In other words, apparent evidence of infection is not necessary to diagnose IRGN, which indicates the possibility of misdiagnosing similar lesions such as C3G as IRGN.

With regard to C3G, typical LM findings show an MPGN pattern characterized by diffuse mesangial and endocapillary proliferative changes with thickening of glomerular capillary walls. On IF findings, C3G was originally characterized by intense glomerular C3 deposition in the absence of C1q, C4, and immunoglobulins, but the modified newer definition allows for weak positivity of some immunoglobulins or other complement components if C3 staining is dominant and at least 2 orders of magnitude greater than any other IF reactant [26,33,34]. The differential diagnosis of C3GN and DDD could be achieved only by EM observation. C3GN shows electron-dense deposits (EDDs) in mesangial, subendothelial, and/or subepithelial spaces [26], in contrast to DDD, which shows characteristic highly osmiophilic EDDs in the intramembranous space [26].

**Figure 1:**
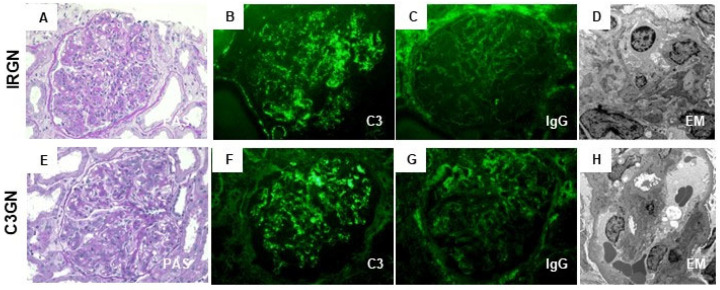
Characteristic pathological findings of C3-dominant infection-related glomerulonephritis (IRGN) and of C3 glomerulonephritis (C3GN). Representative photos of C3-dominant IRGN (**A**–**D**) and C3GN (**E**–**H**) diagnosed in our hospital. (**A**,**E**): The glomerulus shows endocapillary and mesangial proliferative change (periodic acid-Schiff stain; original magnification ×400). (**B**,**F**) immunofluorescence (IF) staining for C3 shows strong positivity, mainly on capillary walls and partially in the mesangial area (original magnification ×400). (**C**,**G**) IF staining for IgG shows weak positivity or negative on capillary walls (original magnification ×400). (**D**,**H**) Electron microscopy shows scattered small-sized subepithelial electron-dense deposits (EDDs) in C3 dominant IRGN and different-size EDDs in the subendothelial and paramesangial area in C3GN (original magnification ×1000).

In fact, pathomorphological findings of IRGN and C3G are generally similar. Specifically, Figure 1A–D shows the characteristic pathology of a case of C3-dominant IRGN (64-year-old man with NRP after onset of phlegmon due to staphylococcal infection) diagnosed in our hospital, which could be indistinguishable from those of our case of C3GN at a glance (Figure 1E–H, 34-year-old woman with chronic GN and persistent reduction in the serum C3 level without any evidence of infection). Moreover, C3GN could show subepithelial EDDs as a hump-shaped pattern on EM, and IRGN occasionally exhibits the MPGN pattern on LM. In short, the location of glomerular EDDs or LM findings alone is insufficient to clearly distinguish these two diseases. We consider that the differential diagnosis of IRGN and C3G according to the diagnostic criteria is still limited.

### 2.2. Differentiation between IRGN and C3G Based on the Classification of MPGN

**Figure 2:**
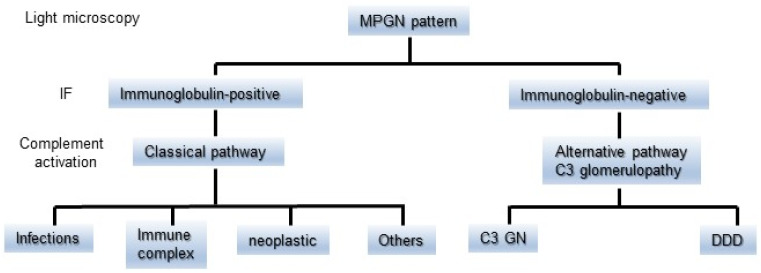
A simple classification of MPGN based on the findings of IF staining and the difference in the state of complement activation. The MPGN pattern detected by light microscopy is divided into immunoglobulin-positive or immunoglobulin-negative on IF staining. It is suspected that immunoglobulin-positive MPGN is induced by classical pathway activation. Immunoglobulin-negative C3-positive MPGN is categorized as C3G due to dysregulation of the alternative pathway. C3G is further subdivided into C3GN and DDD. C3G: C3 glomerulopathy, DDD: dense deposit disease, IF: immunofluorescence, and MPGN: membranoproliferative glomerulonephritis.

**Figure 3:**
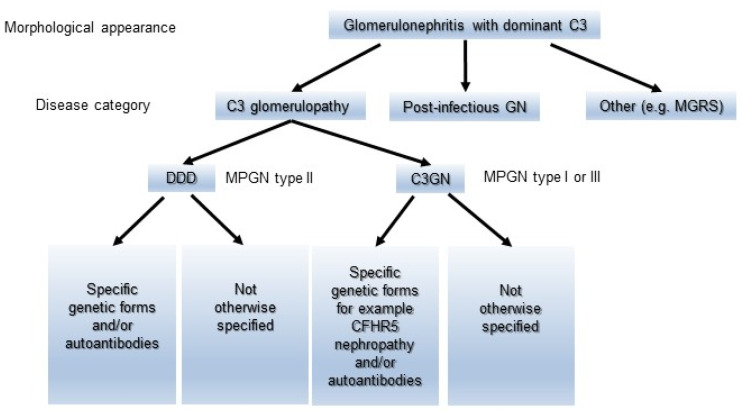
A schematic diagram showing an approach to classifying the morphological changes in glomerulonephritis with dominant C3. The morphological changes are divided into C3G, Postinfectious GN, and MGRS. C3G is further subdivided into C3GN and DDD, and then categorized into each group in detail according to the etiology. C3GN: C3 glomerulonephritis, DDD: dense deposit disease, MGRS: monoclonal gammopathy of renal significance, and Postinfectious GN: postinfectious glomerulonephritis.

MPGN has been classified as types I to III based on the distribution of EDDs on the glomerulus. As a general rule, MPGN types I and III have been characterized as IC diseases showing subendothelial or subendothelial/subepithelial EDDs, and MPGN type II was categorized as DDD with intramembranous EDDs [8,26]. Meanwhile, cases of immunoglobulin-negative MPGN types I and III have been identified for decades. The majority of these types of pathology were ultimately defined as C3G or C3GN [8,26]. Sethi et al. accordingly proposed a simple classification of MPGN (Figure 2) that was based on IF findings and the state of the complement activation system to prevent unnecessary confusion [8]. Immunoglobulin-positive MPGN is recognized to be induced by classical pathway (CP) activation, and a concerted effort should be made to identify the underlying cause of antigenemia. Immunoglobulin-negative C3-positive MPGN is categorized as C3G due to dysregulation of the AP and terminal complement complex. However, their proposal could have the risk of misidentifying C3-dominant IRGN as C3G, and cases of C3G with weakly immunoglobulin deposition in glomeruli might not be definitely diagnosed. Pickering et al. then introduced the term “glomerulonephritis with dominant C3” and a schematic diagram showing an approach to classify the morphological changes in such glomerular lesions, including C3G, PIGN, and monoclonal gammopathy of renal significance (MGRS) (Figure 3). Some MGRS is reported to produce autoantibodies to FH, leading to dysregulation of the AP [11,35]. It is considered that use of the above descriptive morphological term is convenient and helpful to differentiate between PIGN and C3G without confusion if the relationship of infection to the development of GN is evident.

### 2.3. Differentiation between IRGN and C3GN Based on IF-P

Regarding C3-dominant IRGN, frequently seen in patients with PSAGN, the exact pathogenetic mechanism has remained a matter of debate. The conventional pathogenic mechanism of PSAGN had been presumed to be the so-called IC theory, involving glomerular deposition of nephritogenic streptococcal antigen and subsequent in situ formation and/or deposition of circulating ICs [19,22,31,36]. However, immunoglobulin deposition on glomeruli is not always significant in this disease. Recent articles indicated that 30–40% of patients with PSAGN are negative for IgG, although C3 is strongly positive in all patients in glomeruli [19,20]. Such a C3-dominant pattern has been highlighted to date. Fish et al. previously suggested the possibility that IgG positivity on glomeruli could be precluded by intensive C3 positivity [37]. They explained this as follows: C3 reached a detectable level for the IF technique, whereas IgG remained below the threshold level for detection [37]. As an opinion supporting their theory, a peculiar form of glomerular IC deposition in which masked deposits required an antigen-retrieval step for visualization was reported. Previously, membranous-like glomerulopathy with masked IgG kappa deposits [38] and atypical IRGN with masked IgG-kappa hump-like deposits [39] were seen on repeated IF on formalin-fixed, paraffin-embedded tissue sections following pronase digestion (IF-P). In addition, histopathological analysis by Messias et al. explored such masked immunoglobulin deposits in 61 cases of IC-type glomerular diseases by IF-P [40]. Of them, 20 cases showed negative to weak glomerular IgG positivity by routine direct IF staining using frozen tissue (IF-F), and then 18 of the 20 cases turned out to be strongly positive for glomerular IgG on IF-P [40]. Currently, IF-F is the gold standard for detecting glomerular immune deposits. However, it was reported that the presence of large and predominant C3 deposits on glomeruli could lead to weak or negative staining for immunoglobulin in IF-F [38,40,41]. Although strong evidence has not yet been established, the possibility of masked glomerular immunoglobulin deposition using the method with IF-P deserves to be evaluated. In particular, results for the appearance rate of masked immunoglobulin deposition between C3-dominant IRGN and C3GN are of interest, since a difference might be a valuable clue to resolve the confusion at the time of diagnosis or help in differentiation between the two diseases. 

## 3. Complement Activation in IRGN and C3G

### 3.1. Complement Cascade

The complement system consists of three pathways: the CP, the lectin pathway (LP), and the AP. The complement cascade is briefly summarized as follows: the amplification phase follows the initiation phase and segues into the terminal phase. Generally, the CP and LP need to be activated via recognition of pathway-specific triggers, such as pathogen-associated molecular patterns, antigen-antibody complexes, or microbial polysaccharides. Meanwhile, the AP is constitutively active through the spontaneous hydrolysis of a thioester bond on C3 to produce C3(H_2_O). This process is termed tick-over and occurs at a rate of around 1% of total C3 per hour [42,43]. Subsequently, activated C3 in the AP leads to the formation of C3 convertase, C3bBb, by combining with FB through cleavage by factor D. Once C3 is activated, a feedback loop amplifies the initial complement response and activation of the terminal pathway by the generation of C5 convertase (C3bBbC3b). This C5 convertase cleaves C5 into C5a and C5b, starting the generation of C5b-9 or membrane attack complex, which induces cell lysis [13,16,44]. In addition, C5a, like C3a, promotes inflammation as a potent anaphylatoxin [16,44].

The above-mentioned complement activation system plays a pivotal role in the progression of both IRGN and C3G. In IRGN, activation of CP, LP, and AP might be involved, which depends on the clinical stage or pathophysiological state [19]. In C3G, acquired or genetic alterations of the regulatory proteins in the AP are essential for pathogenesis [11,12,35]. In both diseases, hypocomplementemia, mainly a reduction in the serum C3 level, is frequently seen, and the level of serum C3 usually returns to the normal range within a month or so in IRGN, especially in cases of PSAGN. However, some patients with IRGN also demonstrated a persistent reduction in the serum C3 level, which is proposed to be due to the effect of AP dysregulation [3,19].

### 3.2. Complement Profile and Activation in IRGN, Especially in PIGN

According to a recent review [30], activation of the CP in PSAGN is suppressed by chemokine-binding evasins secreted by *Streptococcus* and by proteins on the streptococcal surface that bind a C4b-binding protein [45,46,47]. Evasins are a family of salivary proteins produced in parasitic ticks that are capable of turning off the first steps of an immune response brought about by chemokines [47]. In fact, normal C1q levels are almost universal in PIGN, and there has been research that indicated the suppressive state of CP in PIGN, but glomerular C1q deposition in PIGN is sometimes detected in RB specimens. Thus, it might be important to keep in mind the partial effect of CP on the development of PIGN. Regarding the LP in PIGN, the association of activated LP with the occurrence of PSAGN has been reported [30]. Polymorphisms of the genes in the LP were demonstrated to aggravate the immune reaction [48]. Indeed, a low serum C4 level in patients with PIGN is occasionally seen, which is thought to be the result of activation of the LP. Taken together, activation of the LP, as well as the CP, is not negligible in PIGN. Nevertheless, there is no doubt that the AP is the most important pathway in the pathogenesis of PIGN. Atypical cases of PIGN in which dysregulation of the AP-induced CKD with persistent proteinuria and hematuria were highlighted in observational studies [2,20,31,49].

As a cause of the dysregulation of the AP in PIGN, the focus has been on the effect of autoimmune mechanisms for decades. The aspect of abnormal complement gene variants in PIGN was not emphasized, although several previous studies performed genetic analyses [50,51]. So far, several characteristic autoantibodies have been demonstrated in patients with PIGN. The frequencies of the appearance of representative autoantibodies against components of the complement system in PIGN and C3G are summarized in Table 1. Anti-C1q antibodies were anticipated as one of the key effectors of the activation of the AP in PIGN. Kozyro et al. reported that 8 of 24 children with PIGN had anti-C1q antibodies [52]. C3NeF, known as the autoantibody targeting C3Bb, was also considered the driver for AP dysregulation in PIGN. A recent review article suggested that transient C3NeF generation was the cause of AP activation in PIGN [30]. In addition, Sethi et al. assessed 11 patients who were diagnosed as having PIGN with persistent activation of the AP, and 7 of them were found to have positive activity for C3NeF [49]. However, the presence of the above-mentioned autoantibodies has not provided the crucial breakthrough for understanding the mechanism of the activated AP in PIGN. Recently, Chauvet et al. showed the presence of anti-FB antibodies (mainly IgG1 subclass) in pediatric patients with PIGN as a useful diagnostic marker [29]. In the acute phase of GN, anti-FB autoantibodies were identified in 31 of 34 (91%) children with PIGN and in four of 28 (14%) children with hypocomplementemic C3G. The sensitivity and specificity of the detection of anti-FB antibodies for diagnosing PIGN were 95% and 82%, respectively. Furthermore, the anti-FB autoantibodies were transient and correlated inversely with plasma levels of C3 and correlated directly with levels of C5b-9. Furthermore, the anti-FB autoantibodies amplified the activity of the C3 convertase in AP. The anti-FB autoantibodies did not stabilize the C3 convertase formed on red blood cells, indicating that anti-FB autoantibody is different from C3NeF in pediatric patients with C3G. Although the mechanism of the emergence of anti-FB autoantibodies remains to be elucidated, a genetic predisposition is posited. Collectively, identifying anti-FB autoantibodies provided critical insight into the pathophysiologic mechanism of PIGN. Screening for anti-FB antibodies might be helpful to avoid misdiagnosing IRGN as C3G.

### 3.3. Complement Profile and Activation in C3G

The involvement of genetic drivers is inevitable when discussing the AP activation in C3G. It was reported that approximately 25% of C3G patients carry variants of complement-related genes, including *C3*, *CFB*, *CFH*, *CFI*, and *CFHR5*, which encode C3, FB, FH, Factor I, and complement FHR protein 5, respectively [11,12,35]. According to a recent review, the most commonly identified genetic variant in C3G is rearrangement at the *CFH* locus, creating CFHR fusion genes [12]. These genetic variants drive the continuous activation of AP, resulting in the development of C3G. Thus, etiopathogenetic diagnosis based on genetic analysis is desirable for C3G patients, although performing such analysis might be a high hurdle in resource-constrained settings.

Acquired drivers, that is, autoantibodies to the complement system, could also affect the activation of the AP in C3G. The majority of patients with C3G were positive for some autoantibodies against complement convertase or specific complement proteins that impair normal convertase or protein function [12,35]. As shown in Table 1, C3NeF against C3bBb is the most common, reported in 50–80% of DDD patients and 44–50% of C3GN patients [27,28]. C3NeF stabilizes and prolongs the half-life of this convertase in the amplification phase by protecting C3bBb from FH-mediated decay [55]. Similarly, C5 nephritic factor (C5NeF) is also common. This autoantibody binds to and stabilizes C3bBbC3b, increasing the half-life of the C5 convertase and generation of sC5b–9 [35]. In the previous analysis, C5NeF, rather than DDD, was deeply involved in the pathogenesis of C3GN [54]. C4 nephritic factor (C4NeF), which binds to and stabilizes C4b2a, was also identified in patients with C3G [53]. Furthermore, not only these nephritic factors, but also autoantibodies against FH and FB, were occasionally detected [29,54]. In the previous reports, anti-FH autoantibodies were detected in approximately 10–20% of C3G patients [29,30,56]. It was demonstrated that these autoantibodies ultimately stabilize C3 convertase by impairing FH-mediated decay [56,57]. Therefore, physicians need to make an effort to evaluate autoantibodies to the complement system when diagnosing C3G. Further studies are required to establish a reliable, reproducible method for detecting such autoantibodies in C3G.

## 4. Glomerular NAPlr Deposition and Plasmin Activity in IRGN and C3G

### 4.1. NAPlr as a Key Diagnostic Marker for IRGN

NAPlr [19,20,21,22,23,24] and streptococcal pyrogenic exotoxin B (SPEB) [58] have been identified as proteins causing IRGN. NAPlr is a nephritogenic protein isolated from Group A streptococcus that is homologous to streptococcal glyceraldehyde-3-phosphate dehydrogenase (GAPDH). Although GAPDH is known as a housekeeping gene, bacterial GAPDH has pleiotropic functions, such as energy production, regulation of gene expression, and plasmin-binding capacity [59,60,61]. Plasmin bound with NAPlr is suspected to be protected from its physiological inhibitors in vivo and keep its enzymatic activity. Accordingly, NAPlr deposited in glomeruli binds with plasmin and induces plasmin-dependent glomerular damage in IRGN [19,20,21]. The proteolytic activity of plasmin in glomeruli would induce glomerular injury directly by degrading extracellular matrix proteins and indirectly by activating pro–matrix metalloproteases [20,23]. Moreover, the plasmin is recognized to have a proinflammatory function by activating and accumulating inflammatory cells [20,23].

With regard to SPEB, it was also isolated from Group A streptococci and was regarded as another nephritogenic protein in PSAGN [58]. Since SPEB was reported to localize within the subepithelial hump by immunoelectron microscopy, according to the paper of Batsford et al. [62], it was suspected to mainly induce glomerular damage as the component of IC. However, SPEB was also found to have plasmin-binding capacity and might induce glomerular injury via plasmin activity [2]. Furthermore, our evaluation of double staining for NAPlr and SPEB showed an essentially similar distribution of these proteins in the glomeruli of PSAGN [22], which allowed us to conjecture that these two proteins might cooperate in the pathogenesis of PSAGN.

**Figure 4:**
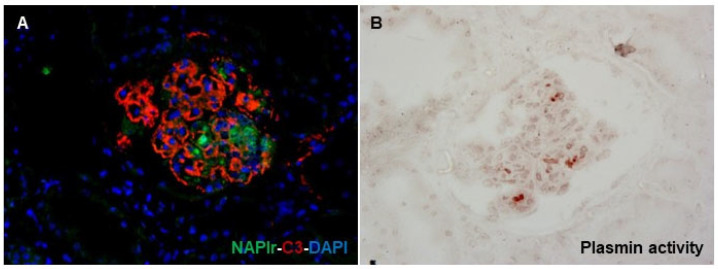
Histological staining for C3, NAPlr, and plasmin activity in the glomeruli of a patient with IRGN induced by streptococci, *Neisseria gonorrhoeae*, and *Chlamydia*. (**A**) Double IF staining for NAPlr (fluorescein isothiocyanate, green) and C3 (Alexa Fluor 594, red) with nuclear staining for DAPI (blue) show glomerular deposition, and different distributions of NAPlr and C3. (**B**) Glomerular plasmin activity assessed by in situ zymography using plasmin-sensitive synthetic peptide on a serial section demonstrates an approximately similar distribution as NAPlr deposition.

Methodologically, glomerular NAPlr deposition is detected by immunostaining, and plasmin activity can be evaluated by zymography using a plasmin-sensitive synthetic substrate [23]. The distributions of NAPlr deposition and plasmin activity in glomeruli, which approximately merge into each other, are seen in the early phase of PSAGN. A previous report demonstrated that RB specimens of PSAGN showed glomerular deposition of NAPlr and plasmin activity in all patients within 2 weeks of disease onset [63]. Furthermore, such glomerular staining or expression patterns have been demonstrated in patients with IRGN caused by infections other than streptococci, such as *Streptococcus pneumoniae* [64], *Aggregatibacter actinomycetemcomitans* [65], *Mycoplasma pneumoniae* [66], and *Staphylococcus aureus* [19,21]. In fact, we also saw a patient with IRGN after the onset of a streptococcal urinary tract infection and sexually transmitted infections by *Neisseria gonorrhoeae* and *Chlamydia* (manuscript in preparation), in which RB specimens clearly demonstrated glomerular NAPlr deposition and plasmin activity in a similar fashion (Figure 4). As mentioned earlier, NAPlr is the same substance as the GAPDH of *Streptococcus*, and GAPDH is universally expressed and may have high homology among various bacteria. Consequently, anti-NAPlr antibodies are likely to cross-react with the GAPDH of bacteria other than *Streptococcus pyogenes*. Therefore, positive glomerular staining for NAPlr and plasmin activity could provide critical histological evidence for the substantial involvement of bacterial infection in the development of GN. As promising candidates as general biomarkers for IRGN, we propose positive glomerular staining for NAPlr and for plasmin activity.

### 4.2. Activation of the AP by NAPlr

The mechanism of overactivation of the AP in IRGN remains to be completely elucidated. Persistent AP activation with a reduction in the serum C3 level, which is apparent in IRGN, is considered to be a factor in the progression to CKD in patients with IRGN. As a possible cause of its activation, the involvement of the aforementioned autoantibodies, such as anti-FB autoantibody [29], has been reported. Furthermore, according to the latest reviews and a previous report [19,20,21,30,67], there is a subpopulation of patients with IRGN having a mild genetic or acquired deficiency in AP regulatory proteins, which is regarded as a trigger for uncontrolled activation of the AP. Yoshizawa et al. demonstrated that NAPlr converted C3 to C3b and induced the formation of iC3b in a dose-dependent manner in human serum samples in vitro [19,24]. Because the glomerular distributions of NAPlr deposition and C3 deposition are essentially different, as shown in Figure 4, the complement AP activation by NAPlr might occur mainly in the circulation (liquid phase) rather than in situ in glomeruli. Direct activation of the AP by NAPlr in the liquid phase may release complement-related anaphylatoxins, including C3a and C5a, resulting in glomerular accumulation of macrophages and neutrophils. However, the evidence for the direct activation of AP by NAPlr in vitro is limited. Further basic research will be indispensable, because this approach might get to the core of the pathogenesis of C3-dominant IRGN.

### 4.3. C3G with Glomerular NAPlr Deposition

Fundamentally, we suggest that positive key diagnostic markers for IRGN are glomerular staining for NAPlr and for plasmin activity. However, positive glomerular staining for these markers was also detected in various glomerular diseases other than IRGN [22]. These cases include C3G [68,69,70,71], MPGN type I [72], antineutrophil cytoplasmic antibody-associated vasculitis [73], and IgA vasculitis [74,75]. No significant difference in staining patterns of NAPlr and plasmin activity among those glomerular diseases and IRGN was detected, which made it hard to differentiate these diseases from IRGN. Moreover, it might be difficult to interpret cases with C3G exhibiting positive glomerular staining for NAPlr and for plasmin activity, despite no apparent history of infection, as recently reported [70]. To the best of our knowledge, four patients diagnosed with C3G with positive glomerular staining for NAPlr and for plasmin activity have been reported in the literature [68,69,70,71]. Table 2 summarizes the critical features of the four previously reported cases. These patients’ ages ranged from pediatric to adolescent. Two of the four had DDD, and the other two had C3GN. In addition, two of the four had a history of preceding infection before biopsy-proven C3G. Unfortunately, the presence of anti-FB autoantibodies was not assessed in all four cases, but two cases evaluated for the presence of C3NeF showed negative results. Furthermore, intriguingly, their renal outcomes were not very poor, although a reduced serum C3 level and mild proteinuria persisted. To uncover the positivity of glomerular NAPlr and plasmin activity in C3G, a future study with a sufficient sample size would be desirable.

## 5. Causal Relationship between IRGN and Development of C3G

The overlapping clinicopathological features of C3G and IRGN, mainly the PIGN pattern, have been an intriguing matter for discussion. In a recent review, Yoshizawa et al. mentioned that pathogenic mechanisms responsible for the development of C3G are essentially the same as the mechanism for C3-dominant PSAGN [19]. As described in this manuscript, several characteristic factors might be useful for the differentiation of these diseases, but there have been several reports that let us presume that C3G and C3-dominant IRGN could just be subtypes of one category of glomerular disease. 

Meanwhile, the causal relationship between C3G and IRGN has been considered as follows. First, preceding infection is suggested. As previously reported [76], a preceding streptococcal infection initially induced PSAGN, but the patient later developed chronic C3G. According to the analysis of Al-Ghaithi et al., 25 (75.8%) of 33 children with PIGN who underwent RB because of an unusual clinical course were ultimately diagnosed as having IRGN, but the remaining 8 (24.2%) were eventually diagnosed as having C3G [77]. Second, persistent causative infection could modify the condition of the host’s complement activation state. Oda et al. mentioned the possibility of alteration of host AP activation due to persistent infection [20]. As shown in Table 2, RB findings in case 3 showed C3G with positive glomerular staining for NAPlr and for plasmin activity, despite the absence of apparent infection [70], which may be due to the modulation of the AP by subclinical infection. Thus, thoroughly assessing the hidden infection by not only bacterial cultures, but also histological staining for NAPlr and for plasmin activity, is critical in cases in which IF findings indicate C3-dominant glomerular deposition. Third, we propose the exacerbation of latent C3G with mild AP dysregulation after the onset of IRGN. Generally, C3G patients show severe clinical presentations such as NRP, but cases of C3G with milder renal clinical manifestations are not rare [15,78]. Environmental triggers of C3G have not been sufficiently documented, but several previous reports suggested that infection might be a trigger for the development of C3G [68,69,76]. Collectively, we propose the following hypothesis: severe infection causes IRGN, followed by the aggravation of non-overt C3G and progression to CKD without healing nephritis. Accumulation of similar cases from around the world and careful analysis are desirable.

## 6. Concluding Remarks

**Figure 5:**
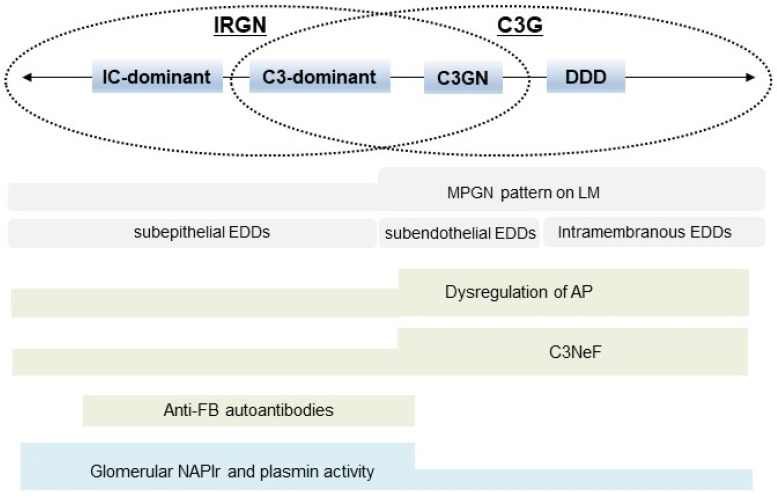
Summary of the concept of this review. Clinico-pathogenic features of IRGN overlap considerably with those of C3G. IRGN: infection-related glomerulonephritis, C3G: C3 glomerulopathy, IC: immune complex, C3GN: C3 glomerulonephritis, DDD: dense deposit disease, MPGN: membranous proliferative glomerulonephritis, LM: light microscopy, EDDs: electron-dense deposits, AP: alternative complement pathway, C3NeF: C3 nephritic factor, FB: factor B, NAPlr: nephritis-associated plasmin receptor, and PA: plasmin activity.

In this review, the characteristic features of IRGN and C3G were described, and the overlapping parts of these two diseases were highlighted. Figure 5 shows a graphic summary of our concept. Clinico-pathogenic features of IRGN overlap considerably with those of C3G, and these two diseases may originally have been categorized into one kidney disease spectrum. However, to select the appropriate treatment strategy, physicians need to differentiate these two diseases as much as possible using information from pathological findings, profiles of components of the complement system, autoantibodies that modify the complement activation state, and immunohistochemical evaluation of infection-related glomerular biomarkers.

## Figures and Tables

**Table 1:** Appearance rate of critical autoantibodies to complement factors in PIGN and C3G.

Autoantibody	PIGN	C3G
C3NeF	Unknown	50–80% [25,27]
C4NeF	Unknown	2–20% [28,53]
C5NeF	Unknown	10–49% [28,54]
Anti-C1q Abs	33% [52]	Unknown
Anti-C3bBb Abs	32% [29]	59% [29]
Anti-C3b Abs	12% [29]	9% [29,54,55]
Anti-FB Abs	91% [29]	14% [29]
Anti-FH Abs	0% [29]	10–21% [29,53]

PIGN = postinfectious glomerulonephritis; C3G = C3 glomerulopathy; Abs = antibodies; FB = factor B; FH = factor H.

**Table 2:** Reported cases with C3G exhibiting positive glomerular staining for NAPlr and for plasmin activity.

	Age (y) /Sex	Diagnosis	NAPlr/PA	Preceding Infection	Anti-FB Autoantibody	C3NeF	Outcomes
Case 1 [68]	12/M	DDD	+/+	Yes	Unmeasured	Unmeasured	NRF with low C3 level 7 years after onset
Case 2 [69]	14/F	DDD	+/+	Yes	Unmeasured	-	NRF with low C3 level 26 months after onset
Case 3 [70]	17/F	C3GN	+/+	No	Unmeasured	-	NRF with low C3 level 5 years after onset
Case 4 [71]	24/F	C3GN	+/ Not inspected	No	Unmeasured	Unmeasured	Persistent renal dysfunction and proteinuria with low C3 level 13 months after onset

C3G = C3 glomerulopathy; NAPlr = nephritis-associated plasmin receptor; FB = factor B; C3NeF = C3 nephritic factor; M = male; F = female; DDD = dense deposit disease; C3GN = C3 glomerulonephritis; NRF = normal renal function.

## Data Availability

The data presented in this study are available upon request.
